# Retraction: MiR-16-5p inhibits breast cancer by reducing AKT3 to restrain NF-kB pathway

**DOI:** 10.1042/BSR-2019-1611_RET

**Published:** 2022-07-27

**Keywords:** AKT3, breast cancer, metastasis, miRNA, NF-κB

This article is being retracted from *Bioscience Reports* at the request of the authors due to technical reasons that they feel unable to address. This Retraction also takes place following receipt of a notification from a reader alerting the Editorial Office to similarities in figure panels across other published papers with no common authors. Figure panels of concern include 2C (common images across papers), 4F (common western blot bands across papers that have been vertically flipped) and 5C/F (common images across papers). The authors are unable to correct the article and wish to retract the article. The Editor-in-Chief and Editorial Board agree with the Retraction.

